# Endocrine Diseases of Newborn: Epidemiology, Pathogenesis, Therapeutic Options, and Outcome “Current Insights Into Disorders of Calcium and Phosphate in the Newborn”

**DOI:** 10.3389/fped.2021.600490

**Published:** 2021-02-05

**Authors:** Tashunka Taylor-Miller, Jeremy Allgrove

**Affiliations:** Department of Endocrinology and Metabolic Medicine, Great Ormond Street Hospital for Children NHS Foundation Trust, London, United Kingdom

**Keywords:** calcium, phosphate, magnesium, vitamin D, PTH, genetics, fibroblast growth factor 23

## Abstract

The physiology and regulation of bone minerals in the fetus and the newborn is significantly different from children and adults. The bone minerals calcium, phosphate and magnesium are all maintained at higher concentrations *in utero* to achieve adequate bone accretion. This is an integral component of normal fetal development which facilitates safe neonatal transition to post-natal life. When deciphering the cause of bone mineral disorders in newborns, the potential differential diagnosis list is broad and complex, including several extremely rare conditions. Also, significant discoveries including new embryological molecular genetic transcription factors, the role of active placental mineral transport, and hormone regulation factors have changed the understanding of calcium and phosphate homeostasis in the fetus and the newborn. This article will guide clinicians through an updated review of calcium and phosphate physiology, then review specific conditions pertinent to successful neonatal care. Furthermore, with the advancement of increasingly rapid molecular genetic testing, genomics will continue to play a greater role in this area of fetal diagnostics and prognostication.

## Take Home Points:

1. Fetal and neonatal mineral metabolism differs significantly from that in later life.
2. The regulation of sodium/phosphate cotransporter activity in the renal tubules is the primary mechanism by which phosphate homeostasis is maintained. Major phosphaturic hormones that regulate renal phosphate handling are PTH and FGF23.
3. Advances in genetics have identified new gene mutations in which have clarified the causes of several conditions previously thought to be “idiopathic.”
4. A thorough understanding of the topic is essential to correct diagnosis and treatment of disorders of calcium and phosphate in the newborn.

## Introduction

Over the past 35 years there have been significant advances in the understanding of materno-fetal mineral homeostatic mechanisms. Parathyroid hormone related peptide (PTHrP) was first described in 1985 as a new compound with parathyroid hormone (PTH)-like bioactivity that accounted for the discrepancy between human umbilical cord and maternal PTH levels (1). This discovery provided new insight as to why fetal PTH levels were so low, yet fetal calcium levels were maintained higher than and independent of maternal calcium concentrations. Another important novel finding was made in 2000, when bone-derived hormone Fibroblast Growth Factor-23 (FGF23) was found to cause autosomal dominant hypophosphataemic rickets (ADHR), which provided the underlying mechanism for the previously unknown “phosphaturic factor” causing hypophosphataemia (2, 3). Genomic discoveries have continued to provide new insights into the mechanisms facilitating transplacental bone mineral transport and unveil the causation of conditions previously thought to be idiopathic. As the fetus accumulates 80% of its bone mineral content in the third gestational trimester (4), this time is critical to achieve normal skeletal mineralisation by 40 weeks gestation and support successful transition to post-natal life. Passive and active transport of bone-minerals occurs across the placenta to achieve higher fetal concentration of calcium, phosphate, and magnesium compared to maternal levels. Once the baby is born, loss of placental delivery of minerals causes a sudden drop in serum concentrations of these bone minerals which triggers a rise in regulating factors such as PTH, 1,25-dihydroxyvitamin D [1,25(OH)_2_D, calcitriol] and FGF23 to maintain postnatal homeostasis. This article will first examine current understanding of fetal-to-neonatal mineral homeostasis mechanisms, and then review specific conditions pertinent to successful neonatal care. Magnesium and vitamin D homeostasis will also be briefly discussed.

### Fetal Calcium Homeostasis

Fetal blood calcium concentrations are maintained ~0.3–0.5 mmol/L higher than in maternal circulation, with the placenta transporting 100–150 mg/kg/day of calcium during the third trimester (4–6). To achieve this, active materno-fetal transplacental transport is facilitated by transmembrane calcium-selective channel TRPV6, calbindin D_9k_ and plasma membrane calcium-ATPase. Once calcium has been delivered to the fetus, concentrations are tightly regulated by the calcium sensing receptor (CaSR) which is primarily expressed in the fetal parathyroid glands and kidneys. The CaSR activates magnesium-dependent G-protein coupled downstream signalling cascades to control PTH secretion and renal calcium handling. Mutations in *CASR* result in distinct phenotypes causing either hyper- or hypocalcaemia.

PTH is integral for achieving normal bone mineralisation and maintaining fetal calcium homeostasis by regulating expression of calciotropic genes and other solute transporters within the placenta (7). By the 10th week of gestation PTH is synthesised from fetal parathyroid glands, but circulating concentrations are kept low during fetal life due to relative hypercalcaemia dictated by the CaSR. Fetal parathyroid glands differentiate from endoderm cells in the third and fourth pharyngeal pouches, and mutations in any of the involved genes or transcription factors results in several genetic hypoparathyroidism conditions (8, 9). Both PTH and PTHrP, acting on PTH1 receptor to increase resorption of calcium from bone and kidney and expression of 1a-hydroxylase enzyme, play a critical role in endochrondral bone formation and stimulation of placental calcium transport (4, 10, 11).

Birth causes disruption of the maternal-fetal calcium supply and rapid 30% drop in serum calcium concentrations (4). This triggers a 2–5-fold increase in PTH secretion to stimulate calcitriol synthesis, resorption of calcium from renal tubules, and mobilisation of calcium from skeletal stores to maintain normocalcaemia in the first 48 postnatal hours (4, 6). Hypocalcaemia can be much more pronounced in premature infants due to lack of third trimester bone mineral accretion and gestational unresponsiveness of the parathyroid glands (6). The gastrointestinal tract then becomes the main source of calcium for the newborn. As such, the feeding mode and volume determines calcium availability. For example, exclusively breast-fed infants receive ~200 mg/day calcium (12). Active calcium absorption is driven by calcitriol. The PTH surge drives upregulation of calcitriol synthesis which increases serum total calcium to adult values within 48 h which are then strictly maintained between 2.12 and 2.62 mmol/L (12). There is evidence, however, that the newborn gut is not fully responsive to calcitriol until 4 weeks of age (4).

### Fetal Phosphorus Homeostasis

Fetal serum phosphate is maintained ~0.5 mmol/L higher in the fetus compared to the mother, though the mechanisms for placental transport are unclear (4). The majority of phosphate, ~60–70 mg/kg/day, is also accumulated during the third trimester of gestation and is stored primarily within bone as hydroxyapatite (5, 6). Phosphate is integral to endochondral bone formation by mediating hypertrophic chondrocyte apoptosis, and in the mineralisation of fetal bone as it is incorporated into the osteoid allowing calcium to bind to it (13).

The homeostatic mechanisms by which phosphate concentrations are “sensed” in humans are not fully understood, though it is possibly via a plasma membrane complex and that intestinal lumen levels are involved in feedback-regulation on renal phosphate reabsorption (14, 15). Elevation of extracellular phosphate activates FGF23, the primary endocrine regulator of phosphate that is produced by osteocytes and osteoblasts in bone. A complex signalling cascade is then activated when FGF23 binds with co-receptor Klotho to the FGF-receptor (FGFR1) in the kidney (16). The three primary actions of FGF23 are: promoting phosphaturia by phosphorylation of the sodium/hydrogen exchange regulatory factor (NHERF1 coded by *SLC9A3R1*) and down-regulation of NaPi type 2a/2c co-transporters in the renal proximal tubule (coded by *SLC34A1* and *SLC34A3*, respectively); reducing calcitriol metabolism by downregulation of 1-alpha-hydroxylase activity and upregulating catabolic enzyme 24-hydroxylase activity; and have a direct effect on parathyroid glands to reduce PTH secretion (14–17). Mutations in any of these genes can cause variable degrees of nephrocalcinosis, hypophosphataemia, hypercalcaemia, and rickets (18–20). There is emerging evidence that phosphate also has a direct effect on the parathyroid glands and CaSR, in that hyperphosphataemia directly inhibits CaSR activity which, in turn, stimulates PTH secretion and thus promotes renal phosphate wasting from the proximal renal tubule (21).

Immediately after birth phosphate concentrations are low, ~2.6 mmol/L, and rise during the first 48 h of life (6). This rise is likely to be due to immature renal excretion mechanisms (4). The main source of phosphate is dietary, so the method of infant feeding will determine phosphate loading. After birth, normal concentrations of phosphate are dependent on growth and must be interpreted within the context of age-related laboratory reference ranges.

### Fetal Magnesium Homeostasis

Like calcium and phosphate, fetal magnesium concentrations *in utero* are also maintained independently of maternal concentrations, only 0.05 mmol/L higher though, and accrual of ~3–5 mg/kg/day primarily occurs in the third trimester gestation (4, 6, 22). Magnesium is absorbed via TRPM6 and TRPM7 transcellular transporters in the gut and renal tubules, however, and the precise mechanisms controlling placental magnesium transfer and fetal homeostasis remain unknown (4). Magnesium is an important cation that binds to the CaSR, causing modest influence on PTH secretion, and hypomagnesemia can blunt effective PTH secretion (23, 24).

### Fetal Vitamin D Homeostasis

Vitamin D plays a much more important role in postnatal life rather than assisting transplacental mineral homeostasis or fetal mineral accretion. Whilst maternal 25-hydroxyvitamin D readily crosses the placenta to achieve fetal concentrations that are 75–100% of maternal concentrations (1), maternal calcitriol does not cross the placenta but is synthesised primarily in fetal kidneys to achieve fetal concentrations 50% that of maternal (4). These low concentrations are likely suppressed by the elevated fetal serum calcium and phosphate, and low concentrations of PTH. However, it has been demonstrated in animal models that the 1,25(OH)_2_D vitamin D receptor (VDR), and thus by proxy calcitriol, is not actually required *in utero* for the fetus to achieve normal calcium, phosphorus, or PTH homeostasis, or for normal skeletal mineralisation as the placenta is providing the required mineral transfer (25, 26). It is after birth that the role of calcitriol becomes vital.

After birth the level of vitamin D (cholecalciferol) intake depends on the mode of feeding as the gut becomes the main source of absorption. Breast milk primarily contains vitamin D in the form of cholecalciferol, as very little 25(OH) vitamin D passes from maternal serum into breast milk. Whilst there is good correlation between maternal cholecalciferol intake and infant serum 25(OH) vitamin D concentrations (27), breast milk contains only a small amount of cholecalciferol – no more than 25 IU/L – which is insufficient to meet newborn daily requirements (28, 29). Though infant formulae are fortified with vitamin D, it is the international consensus guidance that all infants should be supplemented with cholecalciferol 400 IU/day for every infant until 12 months of age regardless of feeding mode (30).

## Neonatal Hypocalcaemia

Neonatal hypocalcaemia is defined in two ways: one, in term and pre-term infants with birth weight >1,500 g as total serum calcium <2.0 mmol/L or ionised calcium <1.1 mmol/L; and two, in pre-term infants with low birth weight (LBW) <1,500 g as a total serum calcium <1.75 mmol/L or ionised calcium <1 mmol/L (31). Clinical signs of hypocalcaemia are difficult to elicit in newborns and hypocalcaemia is often asymptomatic within 72 h of birth. Acute hypocalcaemia can present as apnoea, irritability, jitteriness, muscle cramps, tetany (including laryngospasm), seizures, cardiac arrhythmias, and QT-segment prolongation. Chronic hypocalcaemia can be more subtle, presenting with dental enamel hypoplasia, subcapsular cataracts, cardiomyopathy, congestive cardiac failure, and basal ganglia calcifications.

The causes of neonatal hypocalcaemia are summarised in Table 1. Parathyroid glands can take >48 h to become responsive to the fetal-to-neonatal transition and important causes of hypocalcaemia can be helpfully thought of as early onset (<72 postnatal hours), or late onset (>72 postnatal hours) (31–33). Several genetic mutations have been found to cause Primary Hypoparathyroidism and should be considered when hypocalcaemia lasts >72 h. Isolated causes of hypoparathyroidism include *GMC2* or PTH-gene mutations, autosomal dominant activating *CASR* or *GNA11* mutations, and X-linked *SOX3* mutations (8). Mutations in *CASR* result in distinct phenotypes causing either hyper- or hypocalcaemia. Activating (gain-of-function) *CASR* mutations decrease the set-point of CaSR and PTH is not secreted when low calcium levels would normally trigger PTH release, resulting in Autosomal Dominant Hypocalcaemia (ADH). Clinically this presents as hypocalcaemia associated with an inappropriately normal-high urinary calcium excretion, presumably due to increased activity of the CaSR in the kidney.

**Table 1:** Summary of the various causes of neonatal hypocalcaemia.

**Early hypocalcaemia <72 h life**	**Late hypocalcaemia >72 h life to 10 days of life inclusive**
• Prematurity • Intrauterine growth restriction • Sepsis • Perinatal asphyxia causing cellular damage and release of intracellular phosphate • Iatrogenic: ∘ Transfusions with citrate blood products ∘ Lipid infusions ∘ Loop diuretics • Maternal factors: ∘ Severe Vitamin D deficiency ∘ Pre-eclampsia ∘ Gestational diabetes associated with hypomagnesaemia ∘ Hyperparathyroidism suppressing infant PTH synthesis ∘ Anti-convulsants ∘ High dose antacids	• Vitamin D deficiency due to inadequate synthesis, intake or absorption • Increased phosphate load: ∘ Enteral feeding with cow's milk ∘ Parenteral nutrition • Primary Hypoparathyroidism: ∘ Isolated vs. Syndromic • Secondary Hypoparathyroidism: ∘ Pseudohypoparathyroidism due to renal disease or *GNAS* mutations ∘ Congenital heart disease ∘ Renal disease ∘ Gastrointestinal disease ∘ Critical illness • Osteopetrosis

Hypoparathyroidism can also be associated with more complex syndromes including: 22q Deletion Syndrome; CHARGE association (*CHD7*); Autoimmune polyglandular syndrome type 1 (*AIRE*); Hypoparathyroidism, sensorineural deafness, and renal dysplasia (HDR) syndrome (*GATA3*); mitochondrial disorders; Sanjad-Sakati and Kenney-Caffey syndromes (*TBCE* or *FAM111A*) (34). The most prevalent of these complex syndromes is DiGeorge syndrome (DGS) due to deletions in the chromosome region 22q11 involving the candidate gene *TBX1*, however microdeletions on chromosome 10 (*GATA3* or *NEBL*) may cause similar phenotypes also associated with cardiac abnormalities (35). Hypocalcaemia in DGS is usually transient and due to underdeveloped parathyroid glands, occurring in 60% of patients mostly during the neonatal period (36). Even normocalcaemic DGS infants are likely to have serum calcium concentrations in the lower half of the normal range, so all should be routinely screened for hypocalcaemia (37).

Osteopetrosis is a very rare cause of neonatal hypocalcaemia, where defective osteoclasts are unable to remodel bone. Hypocalcaemic tetany and seizures can be a presenting feature due to the inability to mobilise calcium stores from bone (38). Osteopetrosis is also associated with increased bone mass, fragility fractures, and bone marrow failure (39). It is usually identifiable with plain radiograph with osteosclerosis and “bone-within-bone” appearance on skeletal survey.

## Neonatal Hypercalcaemia

Whilst there is no consensus on definition, neonatal hypercalcaemia may be considered when calcium is greater than two standard deviations above the normal mean (ionised calcium above 1.32 mmol/L or adjusted serum calcium >2.6 mmol/L) (40), or a total serum calcium >2.9 mmol/L (41). Clinical features in the newborn can be difficult to identify and may include polyuria, polydipsia, lethargy, vomiting, abdominal pain, failure to thrive, irritability, and seizures. The causes of hypercalcaemia associated with appropriately suppressed PTH secretion are extensive. Neonatal sepsis is the most common cause that lasts longer than two consecutive days, possibly due to extra-renal macrophage production of calcitriol and/or increased cytokine activity (41). Other important common causes include: subcutaneous fat necrosis where granulomatous inflammatory cells express increased calcitriol; increased calcium and phosphate intake in infants receiving parenteral nutrition; Vitamin D intoxication; and Williams-Beuren Syndrome due to gene deletion on chromosome 7q11.23, which classically present with mild hypercalcaemia (2.9 mmol/L) associated with supravalvar aortic stenosis and distinctive facial features (42).

There has been a number of new genetic discoveries for various causes of neonatal hypercalcaemia. Identification of the active calcium placental transport mechanism, transmembrane calcium-selective channel TRPV6, provides genetic explanation for a condition previously labelled “Transient Neonatal Hyperparathyroidism,” which was thought to be have been caused by congenital vitamin D deficiency. Newly identified compound heterozygous missense mutations in *TRPV6* were found to prevent adequate transplacental calcium transport and cause potentially lethal skeletal abnormalities (undermineralised bone, fractures, periosteal, and metaphyseal changes), elevated PTH, hypomagnesaemia and hypovitaminosis D (33, 43–45). New genetic mutations in vitamin D metabolism and urinary phosphate excretion have also been identified as causes of previously labelled “Idiopathic Infantile Hypercalcaemia.” Loss-of-function mutation in *CYP24A1* [encoding vitamin D breakdown enzyme 25(OH) vitamin D_3_ 24-hydroxylase] and in *SLC34A1* (encoding renal proximal tubular NaPi co-transporter) and NHERF1 (a modifier of *SLC34A1*) cause accumulation of calcitriol, hypercalcaemia, hypercalciuria, and nephrocalcinosis (19, 46). An awareness of these last two conditions is important in reducing long-term kidney disease in adulthood. Other causes of neonatal hypercalcaemia currently do not have an identifiable cause and are truly idiopathic.

When hypercalcaemia is associated with inappropriately detectable PTH (within laboratory “normal” range or elevated), then causes to consider include rare inactivating (loss-of-function) *CASR* gene mutations which result in CaSR insensitivity and thus PTH secretion is not switched-off until higher-than-normal calcium concentrations, causing Familial Hypocalciuric Hypercalcaemia (FHH). Clinically this presents as generally asymptomatic hypercalcaemia, inappropriately detectable concentrations of PTH, associated with reduced renal calcium excretion. The mode of inheritance appears to cause a “dosage effect” with regard to the severity of hypercalcaemia (47). In contrast, homozygous or compound heterozygous loss-of-function *CASR* mutations, or heterozygous mutations where the mother is not affected, cause Neonatal Severe Hyperparathyroidism (NSHPT), which is a severe phenotype that is associated with life-threatening hypercalcaemia, hyperparathyroid bone disease and multiple fractures. Early diagnosis is critical to prevent death or neuromotor delay (48).

## Rickets and Rickets-Like Disorders

Rickets is a disorder of the growth plate resulting from defective chondrocyte apoptosis and osteoid mineralisation. Rickets can be sub-classified as: calciopenic (due to dietary deficiency of vitamin D or calcium, or due to defects of vitamin D metabolism or action); or phosphopenic (due to renal phosphate wasting or deficiency of phosphate intake). A concurrent serum PTH measurement can be useful when distinguishing between calciopenic (PTH should be elevated) and phosphopenic rickets (PTH likely within normal laboratory reference range or only modest elevation). The radiological and clinical features depend on the child's age at presentation and underlying cause, but include metaphyseal widening, under-mineralised bone matrix (osteomalacia), delayed closure of fontanelles, softening of the skull bones (craniotabes), parietal and frontal bone bossing, craniosynostosis, bowing of long bones, pseudofracture (Looser zones) and fractures, sequelae of hypocalcaemia (seizures, tetany, dilated cardiomyopathy), failure to thrive, decreased muscle tone, and delayed motor milestones (30, 48, 49). It is important to note that craniotabes and ulnar cupping can be normal variants seen in healthy neonates without correlation to maternal or neonatal vitamin D concentrations (4).

Nutritional rickets are the most common form of rickets in the newborn period, and consensus guidelines recommend vitamin D sufficiency of 25(OH) vitamin D >50 nmol/L (30). Breastfed infants of vitamin D deficient mothers, especially with darker skin pigmentation, are high risk and should be routinely screened. The resulting hypocalcaemia is exacerbated by an immature newborn PTH-response. There is significant controversy about the appropriateness of the term “congenital rickets,” as mothers were found to have compounding conditions (such as malnutrition or malabsorption) that interfered with vitamin D metabolism. Therefore, “neonatal rickets” is the preferred terminology (4). Genetic mutations in vitamin D synthesis (25-hydroxylase and 1-alpha-hydroxylase deficiencies) or action (VDR mutations) are rarer causes of vitamin D associated rickets. The demand for skeletal mineral delivery is high, especially in preterm babies, and rachitic skeletal changes that are absent at birth can develop rapidly within 16 days after delivery (50).

Hypophosphataemic rickets uncommonly presents in the newborn. Special consideration is needed, however, to ensure adequate enteral or parenteral supplementation meets the increased skeletal mineralisation demands. Feeding with amino-acid elemental formulas, such as Neocate®, and high-dose antacids, has been associated with reduced bioavailability of phosphate resulting in hypophosphataemic rickets and fractures (51, 52). Hemizygous mutations in phosphate-regulating endopeptidase (*PHEX*) gene lead to overexpression of FGF23 and cause X-Linked hypophosphataemic rickets (XLH) (53). While the renal phosphate wasting is present from birth, XLH does not tend to become clinically apparent until child begins to weight-bear.

The differential diagnosis for rachitic-appearing skeletal changes in newborns is large and includes neonatal hyperparathyroidism, skeletal dysplasias, hypophosphatasia, metaphyseal chondrodysplasia, osteogenesis imperfecta (OI), and vitamin C deficiency (Scurvy) (49). Of these, hypophosphatasia caused by loss-of-function *TNSALP* mutations, in its severest forms (perinatal and infantile) can present with profound skeletal hypomineralisation and bone deformity, hypercalcaemia with downregulation of PTH, and hypercalciuria (54).

## Bone Fragility

When considering conditions that present *in utero* and the neonatal period with bone fragility, OI and OI-like disorders may jump to mind. However, they do not tend to present with biochemical mineral disturbance. The most likely cause of fragile or poorly mineralised bones associated with mineral disturbance in the newborn is Metabolic Bone Disease of Prematurity (MBDP), which has multiple contributors to its characteristic biochemical and radiological findings, see Table 2. Clinical features can develop between 3 and 12 weeks of age, so it is important to screen routinely for biochemical evidence of MBDP with serum alkaline phosphatase (ALP), albumin-adjusted calcium, phosphate, and PTH concentrations from 4 weeks of age in at-risk groups (5, 55). In the premature infant gut phosphate is more readily absorbed than calcium so it is important to ascertain whether MBDP is due to hypophosphatemia or hypocalcaemia. A PTH level paired with serum calcium and phosphate, and urine renal tubular resorption of phosphate (TRP) measurement will help differentiate between hypophosphatemia or hypocalcaemia. Secondary elevation of PTH will occur to maintain normocalcaemia, whereas this compensation does not tend to occur with hypophosphatemia and is associated with decreased phosphaturia (56). Initiating the correct supplementation depending on deficiency is important, as phosphate supplementation in the hypocalcaemic state will bind ionised calcium, exacerbating hypocalcaemia, driving PTH higher, exacerbating renal phosphate loss, and worsening MBPD. While there is no biochemical cut-off consensus to diagnose MBDP, in preterm infants <33 weeks of gestation the combination of bone turnover marker ALP >900 IU/L and phosphate <1.8 mmol/L is associated with sensitivity of 70% and specificity of 100% of having low bone mineral density at 3 months corrected age (57). In practice, lower threshold ALP 500–800 IU/L in infants <34 weeks of gestation is used to implement supplementation (58, 59).

**Table 2:** Factors contributing to Metabolic Bone Disease of Prematurity.

**Antenatal factors**	**Postnatal factors**
• Prematurity <34 weeks of gestation • LBW <1,500 grams • Pathological condition inhibiting macro- and micronutrient placental transfer (chorioamnionitis, pre-eclampsia, intrauterine growth restriction)	• Necrotising enterocolitis • Liver or Renal disease • Late establishment of enteral feeds/prolonged total PN >4 weeks • Chronic lung disease/ bronchopulmonary dysplasia • Medications causing bone resorption (loop diuretics, glucocorticoids) • Antacids • Methylxanthines (caffeine for apnoea of prematurity)

Radiological changes occur late as bones will only appear generally osteopenic on X-ray when >20% of bone mineral is lost (60). Fragility fractures can occur with incidence reported between 17 and 34%, usually after 10 weeks of age in long bones or ribs up until 6 months of uncorrected gestational age (5). Although all fractures are painful, rib fractures often remain undetected by parents and clinical staff until found incidentally on routine chest X-ray (61). Routine screening with X-ray for MBDP is not indicated in the absence of biochemical disease. Techniques such as dual energy X-ray absorptiometry, peripheral quantitative computed tomography, and quantitative ultrasound have been utilised in research settings (60). There are, however, no normative data <5 years of age, which limits their widespread clinical use.

Prevention of MBDP is limited by multiple factors including the inability of premature infants to tolerate full enteral feeding volumes, the inability to deliver high-dose calcium and phosphate via parenteral nutrition (PN) due to solubility and precipitation limits, and reduced gut absorption of calcium and phosphate. The recommended range delivered via PN route is calcium 40–120 mg/kg/day (1.3–3 mmol/kg/day) and phosphate 31–71 mg/kg/day (1–2.3 mmol/kg/day), and via enteral route is calcium 120–200 mg/kg/day and phosphate 60–140 mg/kg/day (62, 63). Enteral calcium and phosphate supplementation should not be given simultaneously or with milk-meals, to avoid precipitation (56). Effective treatment of MBDP should include routine supplementation with cholecalciferol once on full enteral feeding, aiming for serum 25(OH) vitamin D >50 nmol/L (56, 58). Once treatment of MBDP has been initiated ongoing monitoring of ALP, albumin-adjusted calcium, phosphate, serum creatinine levels, and urine creatinine and TRP is important to avoid hypercalcaemia, hyperphosphataemia, and nephrocalcinosis.

### Investigations to Arrange

Essential investigations to assess calcium and phosphate homeostasis include concurrent serum calcium, phosphate, magnesium, PTH, albumin, ALP, electrolytes and renal function, and 25-hydroxyvitamin D levels. Most laboratories provide a calcium corrected for albumin concentration, if not then the following formula will give you the albumin-adjusted serum calcium mmol/L: measured total serum calcium mmol/L + 0.02 × (40 gm/L – measured serum albumin gm/L). High risk infants such as LBW, prematurity <34 weeks, infant of diabetic mother, and prenatal asphyxia should be routinely screened for hypocalcaemia within the first 48 h of age.

If neonatal blood quantities are particularly scarce, then a capillary or venous blood gas will give an ionised calcium value, and serum phosphate can be used as an indirect marker of PTH activity (i.e., low phosphate concentrations reflect high PTH activity and vice versa). Additional investigations such as 1,25(OH)_2_D and serum DNA for genetic analysis may be required but this should follow discussion with local Paediatric Endocrinology service.

Urinary electrolytes and glucose should be part of routine analysis, to calculate renal calcium: creatinine ratio, and to assess renal tubular function.

Radiology should be considered in specific circumstances, namely when bone mineralisation or skeletal dysplasia is a concern. Usually, diagnosis can be made on a limited series to reduce the neonate's radiation exposure including plain films of anterior-posterior chest and metaphysis of a long bone (e.g., unilateral distal femur or wrist). Specific skull films (looking for Wormian bones) or a full skeletal survey are required if bone fragility or skeletal dysplasia are a concern. The need for these more extensive investigations should be discussed with a local specialist paediatric Radiologist.

## Treatment

Appropriate treatment depends on the cause. Where the primary cause is mineral deficiency, additional supplements of calcium, phosphate and, if necessary, magnesium should be given. This can usually be achieved orally but, if demineralisation is severe then intravenous infusion may be required. Vitamin D deficiency should always be corrected.

Hypoparathyroidism, particularly if symptomatic, may require treatment with vitamin D analogues calcitriol or its prohormone alfacalcidol, but caution must be taken to ensure that hypercalciuria and nephrocalcinosis do not result from this treatment. New treatment options in the form of subcutaneous injections of synthetic human PTH teriparatide (hPTH 1-34) and recombinant human PTH (rhPTH 1-84) have been used, particularly where activating mutations of CaSR are the cause of the hypoparathyroidism (23, 64, 65). Calcilytic agents, which reduce the sensitivity of CaSR, are also being investigated as novel therapies for activating CaSR mutations (66).

Hyperparathyroidism can sometimes be corrected with the use of bisphosphonate therapy (although this can lead to an increase in PTH secretion) and/or calcimimetic agents such as cinacalcet (which activate the CaSR, thus increasing the receptor's sensitivity and reducing PTH secretion). Total parathyroidectomy may occasionally be required for intractable NSHPT. Burosumab, a monoclonal antibody to FGF23, is not yet licensed for infants with XLH under 1 year of age but is available in the United Kingdom under an Early Access to Medicines scheme.

## Role of Genomics

Genetics plays an important part in diagnosis as an increasing proportion of cases have been found to have a genetic basis. Liaison with a clinical geneticist can be invaluable.

## Conclusion

The physiology of mineral metabolism differs considerably between fetal and post-natal life. The neonatal period is one of transition from one to the other and a thorough understanding of these processes is required to be able to diagnose and treat the various conditions when they arise.

## Author Contributions

TT-M: writing—original draft, review, and editing. JA: conceptualisation, supervision, writing—review, and editing. All authors have approved the final version of the manuscript.

## Conflict of Interest

The authors declare that the research was conducted in the absence of any commercial or financial relationships that could be construed as a potential conflict of interest.
